# NUT Carcinoma Arising from the Parotid Gland: A Case Report and Review of the Literature

**DOI:** 10.1007/s12105-020-01254-9

**Published:** 2020-12-22

**Authors:** Wei-Ning Saik, Philip Da Forno, Khin Thway, Syed Ali Khurram

**Affiliations:** 1 Unit of Oral and Maxillofacial Pathology, School of Clinical Dentistry, 19 Claremont Crescent, Sheffield, S10 2TA UK; 2 Department of Histopathology, Leicester Royal Infirmary, University Hospitals of Leicester, Leicester, UK; 3 Head and Neck Unit; Sarcoma Unit, The Royal Marsden NHS Foundation Trust, London, UK

**Keywords:** NUT carcinoma, *BRD4-NUT*, Parotid gland, Salivary gland, Poorly differentiated carcinoma, Head and neck

## Abstract

NUT carcinoma is an aggressive carcinoma with an overall poor survival outcome. The mediastinum and head and neck area, especially the sinonasal region, are among the common sites of disease. Histopathological diagnosis of NUT carcinoma is often very challenging due to its overlapping features with other poorly differentiated carcinomas. We report a case of NUT carcinoma arising from the parotid gland of a young female patient. Primary NUT carcinoma of salivary gland is very rare, with only 15 such cases reported in the literature to date. Our case highlights the diagnostic challenges associated with such lesions.

## Introduction

NUT carcinoma is a rare, poorly differentiated carcinoma characterised by a genomic rearrangement of the nuclear protein of testis (NUT) gene, with *BRD4-NUT* being the most common fusion variant. It generally arises from the mediastinum, but numerous cases involving the head and neck, mainly from the sinonasal region, have been reported [1–5]. NUT carcinoma has a poor prognosis, with a mean survival of less than 12 months from diagnosis. People of all ages are equally affected, with no gender predilection [6–9]. Patients normally present with advanced lesions, typically of a rapidly expanding mass at the site involved [6]. There are no effective treatment regimens available to date.

## Case History

**Fig. 1:**
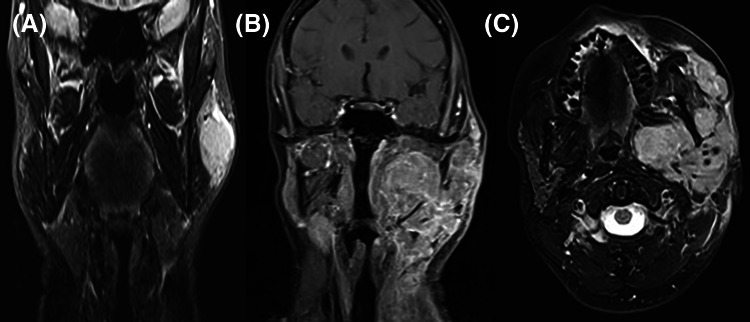
T2-weighted MRI images from left to right: **a** Pre-operative staging image in the coronal plane demonstrates a well-defined tumour in the left parotid gland. **b**, **c** Imaging one month later demonstrates extensive tumour invading into the lower neck, mandible and skull base in both coronal and axial planes

A 34-year-old 6-week post-partum female with no significant medical history presented with a short-term history of a left parotid lump. Magnetic resonance imaging (MRI) and positron emission tomography (PET) imaging identified a solitary well-defined, 38 mm lesion within the left parotid gland (Fig. 1). The initial core biopsy was suggestive of a high-grade neuroendocrine carcinoma. Following this, the patient underwent a radical parotidectomy and ipsilateral selective neck dissection. The histology of the left parotidectomy was reported locally as a high-grade salivary gland neoplasm, possibly high-grade mucoepidermoid carcinoma. There was evidence of perineural invasion as well as metastatic deposits in two lymph nodes. The patient underwent adjuvant radiotherapy; however, a new lump appeared one month later, at the left parotidectomy surgical site. MRI and Computerized tomography (CT) imaging demonstrated a recurrent lesion in the left parotid region, accompanied by widespread metastatic disease in the spine and skull base (Fig. 1).

**Fig. 2:**
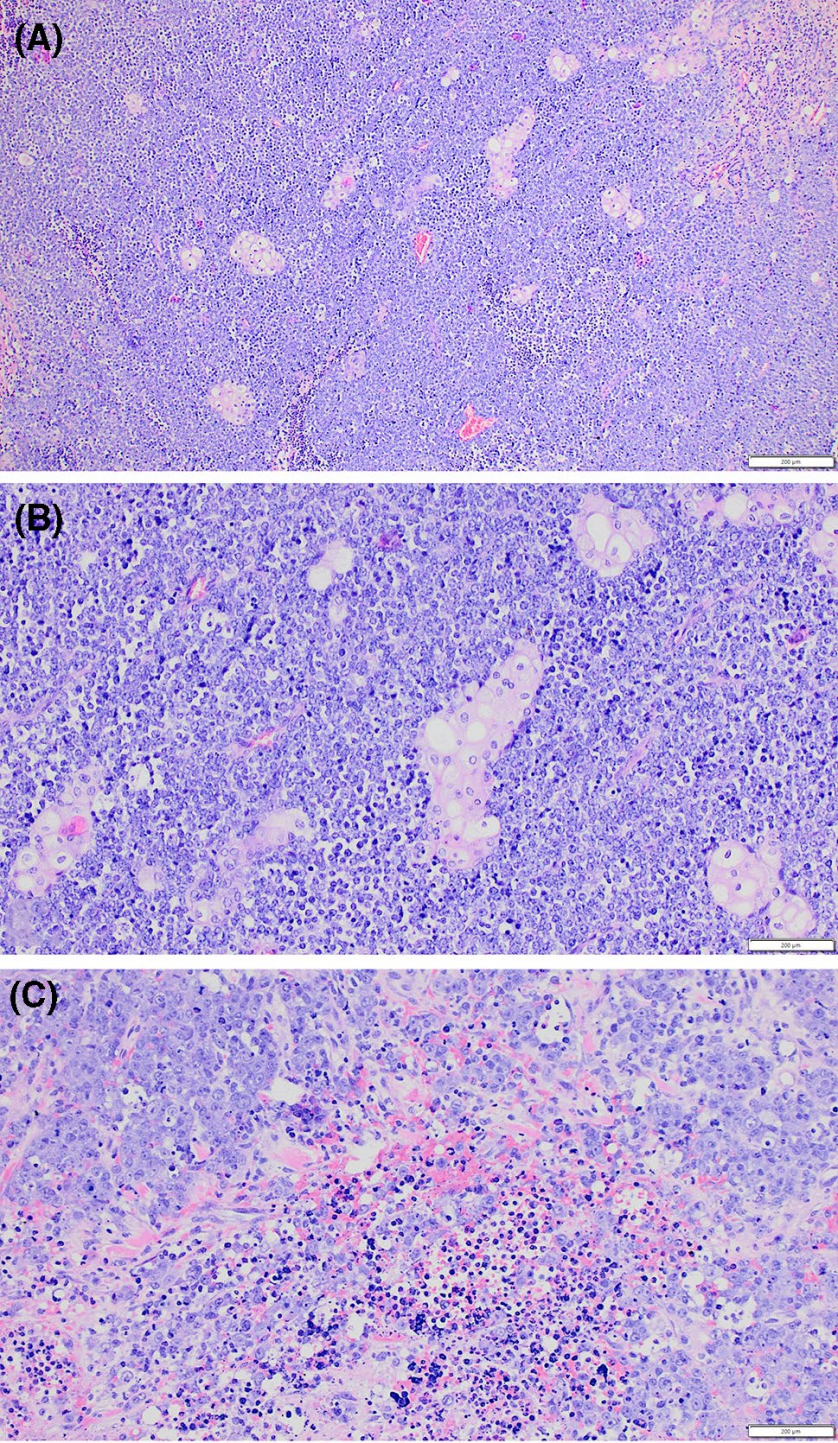
Representative H&E photomicrographs showing sheets of the carcinoma with a small round blue cell appearance. Note the abrupt squamous differentiation (**a**, **b**) and necrosis (**c**)

Following this recurrence, the pathology was reviewed at a tertiary specialist oral and maxillofacial pathology department. Histology showed a multilobular and widely infiltrative neoplasm largely with a ‘small round blue cell’ morphology, exhibiting largely cohesive sheets of undifferentiated cells (Fig. 2). The cells demonstrated significant nuclear and cellular pleomorphism, with prominent nucleoli with focal cytoplasmic clearing. This was accompanied by extensive necrosis and frequent mitoses. Focally, there was evidence of an abrupt squamous appearance (Fig. 2). Extraglandular invasion was present, with tumour islands extending into the surrounding skeletal muscle, in addition to extensive perineural invasion and lymphovascular invasion. There was metastatic disease in one periparotid lymph node and a facial node, with extranodal extension in the latter.

Immunohistochemistry (IHC) showed strong and diffuse expression of p16, p63 and p40, whereas more variable staining was observed for AE1/AE3, CK5/6, Cam 5.2 and CD56. CK7 expression was only seen in the squamous/epithelial islands with a large proportion of the neoplastic population being negative. There was focal expression of synaptophysin, whereas CK14, CK20, chromogranin, CD45, CD99, S100, SMA, TTF1, AR, ER, HER-2 and EBER ISH were negative. Fluorescence in-situ hybridization (FISH) with *MAML2* break-apart probe) showed no evidence of *MAML2* rearrangement.

**Fig. 3:**
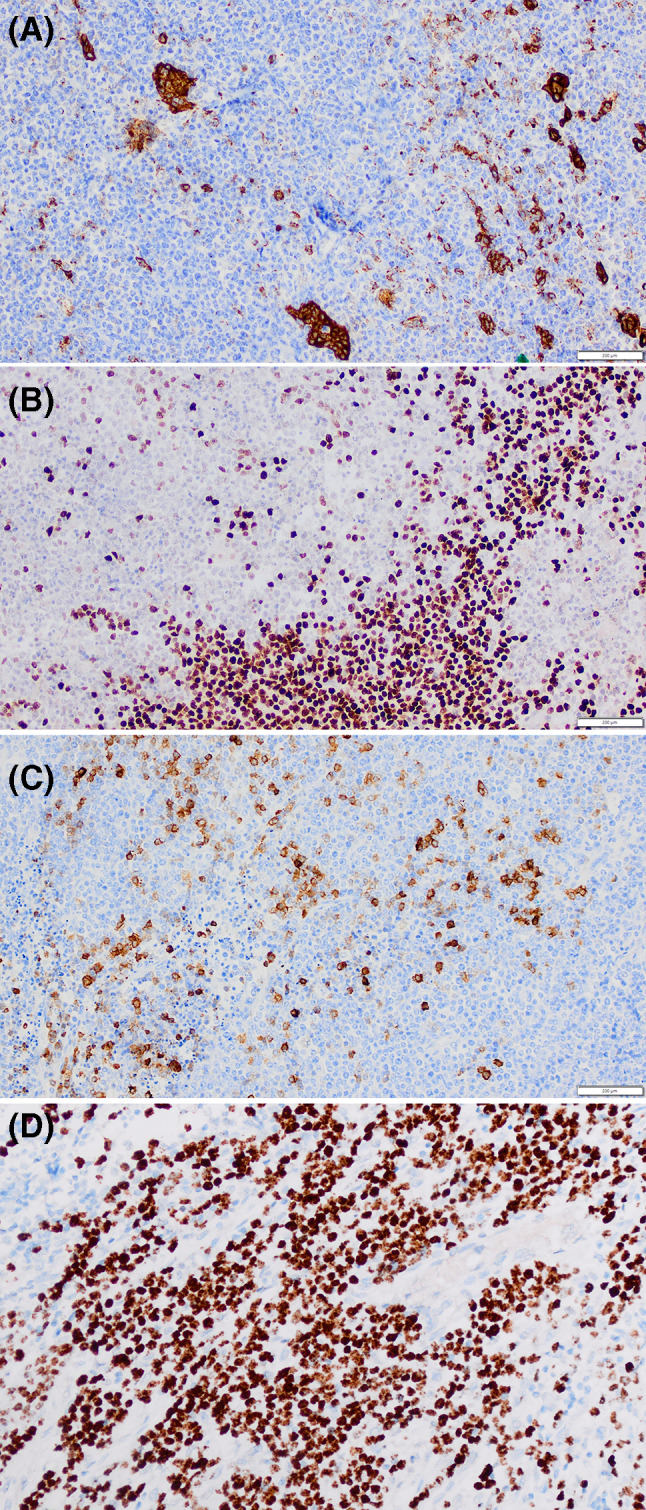
Representative photomicrographs showing IHC staining for AE1/AE3 (**a**), p40 (**b**), synaptophysin (**c**) and NUT (**d**)

The diagnosis of NUT carcinoma was confirmed following strong and diffuse nuclear staining for NUT throughout the tumoral population (Fig. 3). Three-weekly Pembrolizumab immunotherapy was administered alongside palliative radiotherapy to the head and neck region and spine. The patient died of progressive and widespread metastatic disease from the original salivary lesion within six months of diagnosis.

## Discussion

NUT carcinoma is considered by some authors to represent a subtype of squamous cell carcinoma (SCC). However, the *BRD4-NUT* fusion and the lack of SCC molecular signature in NUT carcinoma makes this relationship somewhat controversial [2, 10]. NUT carcinoma has overlapping features with other poorly differentiated carcinomas, making the diagnosis challenging, and extensive IHC and molecular testing are often required for definitive diagnosis. Although the number of reported salivary gland origin NUT carcinomas is low, it is thought that the prevalence of this disease remains under-reported due to misdiagnosis [7, 11–13].

Characteristic histopathological features of NUT carcinoma include diffuse sheets of undifferentiated oval-to-polygonal cells with prominent nucleoli showing eosinophilic-to-clear cytoplasm. This is accompanied by necrosis, mitoses and abrupt keratinisation or squamous change. Recognizing these features can be helpful to exclude some other neoplasms, but these features are not entirely diagnostic. NUT carcinomas arising from the salivary glands are known to demonstrate a similar immunoprofile to those seen in other anatomic sites. Moreover, diagnosis is likely to be challenging on core biopsy material, which may not be representative of the complete morphology and architecture of the neoplasm. The tumour commonly expresses cytokeratins and squamous markers such as p63. The morphology of NUT carcinoma can often mimic neuroendocrine carcinoma; however, neuroendocrine markers are predominantly negative, with variable patchy positivity for synaptophysin and CD56 being described [8, 10, 14]. This variable expression of synaptophysin is a major pitfall, in particular on biopsies.

This case was challenging because the largely undifferentiated morphological appearance was difficult to distinguish from a high-grade salivary neoplasm (e.g. neuroendocrine carcinoma, high grade MEC, acinic cell carcinoma with high-grade transformation, high grade myoepithelial carcinoma) or a metastatic neoplasm [15, 16]. Salivary gland neoplasms that commonly show a clear cell population such as mucoepidermoid carcinoma, epithelial-myoepithelial carcinoma and myoepithelial carcinoma should be considered in the differential diagnosis, although it is uncommon to see undifferentiated or very high-grade features in these tumours. The presence of ‘squamous’ or ‘epidermoid’ areas and clear cells mimicking mucoepidermoid carcinoma can be potentially misleading; however, the absence of mucin, cytokeratin 7 expression and lack of *MAML2* rearrangement can help exclude this possibility. Clear cell change can easily be mistaken for sebaceous differentiation and can be ruled out using androgen receptor. Staining for p16 is positive in approximately 40% of NUT carcinoma, which can suggest a diagnosis of metastatic oropharyngeal SCC. Crucially, however, tests for HPV will be negative, and clinical examination and imaging will not show an oropharyngeal lesion. The absence of convincing neuroendocrine marker expression is not sufficient for the diagnosis of neuroendocrine carcinomas, and the absence of p40 and CD99 positivity helps rule out Ewing Sarcoma whereas molecular evidence of *EWSR1* gene rearrangement aids in the exclusion of adamantinoma like Ewing sarcoma as these can show consistent cytokeratin and p40 expression [10, 11, 17].

Confirming the diagnosis of NUT carcinoma is not possible without the use of NUT IHC or molecular testing. IHC for NUT is performed using commercially available NUT (C52B1) antibody, which shows 100% specificity and 87% sensitivity. This IHC has been shown to be more sensitive than other methods, with diffuse and strong expression in more than 50% of tumour nuclei confirming the diagnosis [18]. Alternative methods to confirm NUT rearrangement include FISH, reverse transcription-polymerase chain reaction (RT-PCR), cytogenetics or next-generation sequencing-based approaches. These methods can help determine the fusion partner (which can include *BRD3*, and more rarely *ZNF532, ZNF592* or *CIC*) and *NSD3* may aid treatment involving targeted inhibitors [7, 19–22]. However, aberrant NUT protein expression can be seen in germ cell neoplasia and must be interpreted cautiously.

NUT carcinoma has an aggressive behaviour, with a poor prognosis. Patients normally present with a rapidly enlarged mass and lymphadenopathy [6, 18]. Currently, there are no gold standards or guidelines for the management of NUT carcinoma. Surgical resection of the primary tumour remains the mainstay of treatment for local disease control. Chemo-radiotherapy can show mixed results, and a limited response to chemotherapy has also been reported. Post-treatment metastatic disease remains a common complication, despite multimodality treatment approaches being adopted in many cases. The development of BET inhibitors aimed to inhibit the binding of *BRD-NUT* and chromatin acetylation with histone deacetylase may provide targeted therapy and potentially improve prognosis for this highly aggressive malignancy [4, 6, 7, 18–20].

## Conclusion

Primary NUT carcinoma originating from the salivary glands is very rare and a diagnostic challenge. This case highlights the importance of considering NUT carcinoma in the provisional diagnosis for undifferentiated or poorly differentiated salivary gland neoplasms. Immunohistochemistry and molecular testing for NUT carcinoma can aid diagnosis; however, the lack of widespread accessibility to these tests can be a potential barrier to diagnosis and management.
